# Advanced strategies for detecting acid sphingomyelinase deficiency type B with attenuated phenotypes

**DOI:** 10.1186/s13023-025-03746-9

**Published:** 2025-05-26

**Authors:** Thomas Villeneuve, Thibaut Jamme, Robin Schwob, Thierry Levade, Grégoire Prévot

**Affiliations:** 1 Respiratory Medicine Department, University Hospital, Toulouse, France; 2 Toulouse Institute for Infectious and Inflammatory Diseases (Infinity), INSERM U1291, CNRS U5282, University Toulouse III, Toulouse, France; 3 Clinical Biochemistry Laboratory, Reference Center for Inherited Metabolic Diseases, Federative Institute of Biology, University Hospital, Toulouse, France; 4 Digital and Data Management Department University Hospital, Toulouse, France; 5 Institute of Research in Computer Science of Toulouse - CNRS UMR5505, University Toulouse III, Toulouse, France; 6 Cancer Research Center of Toulouse (CRCT), INSERM UMR1037, University Toulouse III, Toulouse, France

**Keywords:** Rare disease diagnosis, Metabolic disease, ASMD type B, Niemann pick B disease

## Abstract

**Background:**

Acid Sphingomyelinase Deficiency (ASMD) type B is a rare lysosomal disorder caused by SMPD1 mutations. Due to its low prevalence and clinical heterogeneity, diagnosis is challenging, and detection is crucial for the initiation of enzyme replacement therapy.

**Methods:**

We conducted a retrospective study (RnIPH 2024-85) at Toulouse University Hospital, analyzing 359,802 lipid profiles (2012–2023). We identified individuals with a total cholesterol/HDL cholesterol ratio > 4.5. A regex-based extraction method screened records for consanguinity, hepatomegaly, splenomegaly, and ground-glass opacities (GGOs), while we also analyzed thrombocytopenia (< 150 × 10⁹/L). Patients meeting ≥ 4/5 criteria underwent clinical review.

**Results:**

Among 63,653 patients with dyslipidemia, 20.3% had thrombocytopenia, 4.93% hepatosplenomegaly, 2.29% GGOs, and 0.24% consanguinity. In total, 179 patients met ≥ 4/5 criteria. Nineteen (10.6%) were pediatric. Three previously diagnosed ASMD type B patients in our center were identified. Additionally, among other conditions, 46 cases (25.7%) had monogenic diseases, and five undiagnosed patients were flagged for ASMD screening.

**Conclusion:**

Our hybrid screening effectively identified ASMD type B cases and potential candidates for genetic testing. This approach combining algorithmic filtering and clinical expertise, could enhance ASMD type B diagnosis.

## Background

Acid Sphingomyelinase Deficiency (ASMD) type B is a non-neuronopathic lysosomal storage disorder caused by deficient acid sphingomyelinase activity due to biallelic pathogenic variants in the *SMPD1* (*Sphingomyelin Phosphodiesterase 1*) gene [1, 2]. ASMD follows an autosomal recessive pattern of inheritance, with an extremely low birth prevalence, estimated at approximately 0.5–1 per 100,000 live births [1, 2]. The predominant clinical manifestations of ASMD type B include hepatosplenomegaly (~ 90%) and interstitial lung disease with ground-glass opacities (~ 80%), while neurological involvement is variable, occurring in approximately 30% of cases [3]. Among the most frequently associated biological abnormalities, dyslipidemia is a hallmark feature, characterized by an elevated cholesterol risk ratio (i.e., total cholesterol/high-density lipoprotein [HDL] cholesterol ratio), observed in both childhood-onset and adult-onset forms [4]. Additionally, thrombocytopenia is present in approximately 50% of affected individuals. Due to its low prevalence, limited awareness, and clinical heterogeneity, ASMD type B remains challenging to diagnose [2]. Improving patient identification is particularly crucial given the recent success of enzyme replacement therapy with olipudase alfa, which has demonstrated sustained long-term efficacy in improving lung function and reducing spleen and liver volume in both pediatric and adult populations [5, 6]. We hypothesize that advanced data extraction techniques could enable large-scale analyses, improving ASMD type B case identification and providing a novel strategy for detecting patients with attenuated phenotypes.

## Methods

This retrospective study, conducted at Toulouse University Hospital (RnIPH 2024-85), is exempt from Ethics Committee submission under French law and complies with MR-004 methodology and GDPR regulations (EU 2016/679). It is registered with CNIL (2206723 v 0), ensuring full adherence to ethical standards. Using the hospital’s databases, we extracted patient data from January 1, 2012, to December 31, 2023, focusing on individuals who underwent lipid profile examinations. In the second phase of our analysis, we refined the dataset by identifying patients with i) a cholesterol risk ratio (total cholesterol/HDL cholesterol) exceeding 4.5. We then applied an additional filtering criterion ii), selecting only those with thrombocytopenia, defined as a platelet count below 150 × 10⁹/L. To enhance case identification, we employed a regular expression (regex)-based extraction method to identify medical records containing specific terms: iii) “consanguinity”, iv) “ground-glass opacities” (GGOs), v) “hepatomegaly” and “splenomegaly.” These criteria were systematically analyzed across medical records and imaging reports. All extracted cases were cross-referenced and analyzed to identify patients meeting the highest number of predefined criteria, specifically those exhibiting at least four out of five key features. For patients fulfilling these criteria, we conducted a detailed clinical record review from a clinician’s perspective to ensure accurate case identification.

## Results

**Fig. 1:**
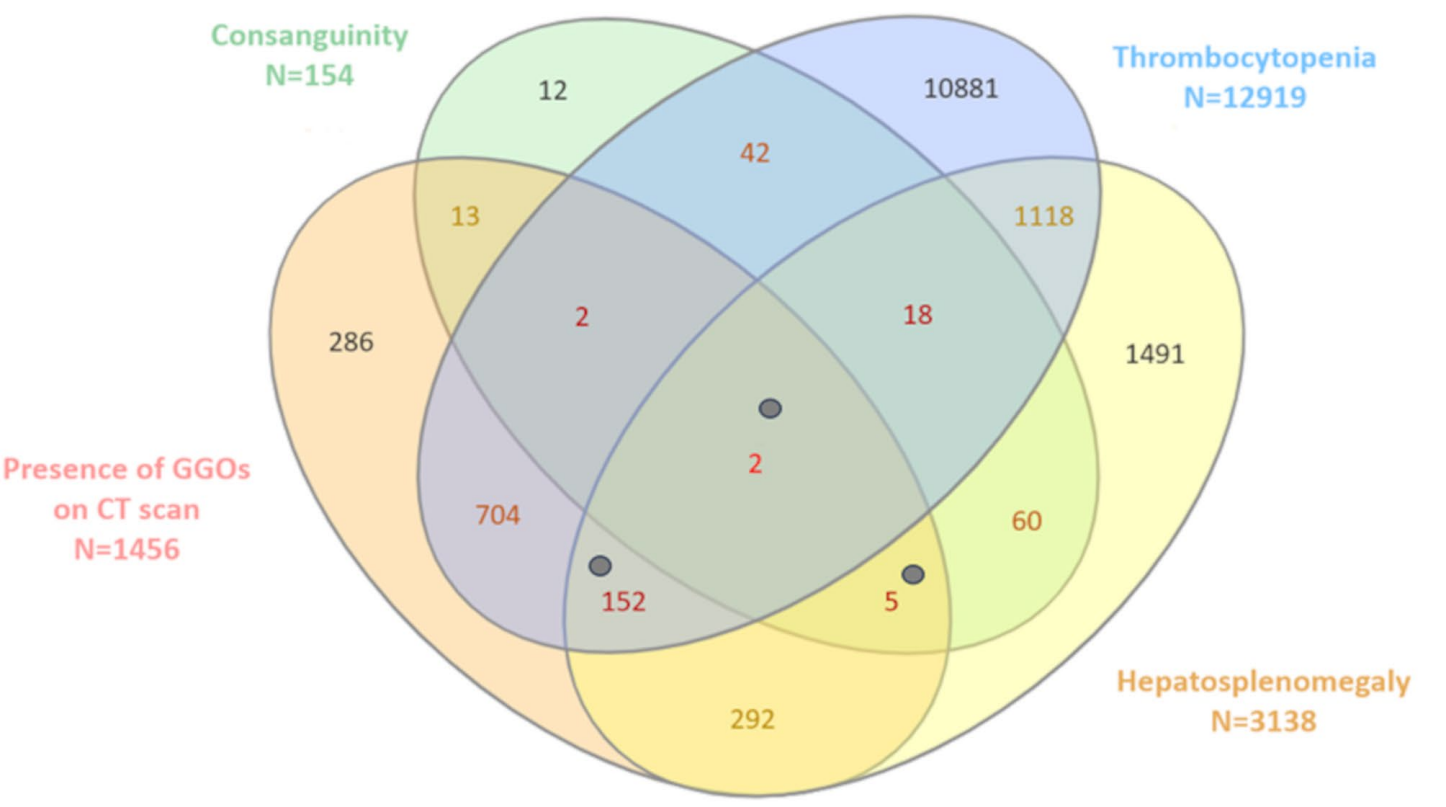
The Venn diagram illustrates the overlap between all types of conditions compatible with ASMD among selected patients with a cholesterol risk ratio > 4.5 (*n* = 63 653). The numbers represent the cohort of patients derived from the total population. Individuals with a confirmed diagnosis of ASMD who met four or five diagnostic criteria are represented by grey points. *Abbreviations*: CT: Computed Tomography; GGOs: Ground-Glass Opacities

A total of 359,802 biological data entries with serum lipid profiles were screened, among which 63,653 patients (17.7%) exhibited a cholesterol risk ratio > 4.5. Among these patients, key clinical manifestations were identified in medical records, including thrombocytopenia in 20.3% (12,919 patients), hepatosplenomegaly in 4.93% (3,138 patients), GGOs on CT (Computed Tomography) scans in 2.29% (1,456 patients), and consanguinity in 0.24% (154 patients). Figure 1 presents a Venn diagram illustrating the distribution and overlap of these clinical features, providing a detailed analysis of shared diagnostic criteria. Following further refinement, 179 patients met at least four out of five predefined diagnostic criteria, suggesting a high probability of ASMD or related metabolic conditions. Among these, 32 patients (17.9%) were deceased at the time of the analysis, while 19 cases (10.6%) were pediatric. The mean age of the cohort was 54.68 years (SD ± 22.76), with a male-to-female ratio (M/F) of 1.98. A diverse spectrum of diseases was identified within the screened cohort. Of these, 46 cases (25.7%) were attributed to rare monogenic disorders (e.g., cystic fibrosis, ciliopathies, Wilson disease) or congenital malformation syndromes (e.g., Shwachman-Diamond syndrome, leprechaunism, arthrogryposis multiplex congenita, Kabuki syndrome). Additionally, 40 cases (22.3%) involved complications related to pre- and post-organ transplantation (e.g., preexisting conditions, opportunistic infections), while 37 cases (20.7%) were associated with systemic inflammatory or autoimmune diseases (e.g., scleroderma, ANCA-associated vasculitis). Chronic interstitial lung diseases accounted for 29 cases (16.2%) (e.g., connective tissue disease-associated interstitial lung disease), whereas cirrhosis was observed in 20 cases (11.7%) (e.g., alcohol-related cirrhosis, non-alcoholic steatohepatitis). Furthermore, 11 cases (6.1%) presented with complications related to HIV infection (e.g., opportunistic infections). Comprehensive genetic testing was performed in 56 patients (31.3%), including 26 cases (14.5%) associated with malformation syndromes. Among patients meeting ≥ 4/5 predefined diagnostic criteria, 12 cases (6.7%) remained undiagnosed, while five potential candidates for ASMD genetic screening were identified within these unrecognized cases. For metabolic disorders, targeted screening was performed on 9 patients (5%), including 6 cases (3.2%) tested for ASMD. Among them, three were confirmed to have ASMD type B (represented as grey points in Fig. 1). Additionally, no cases of Gaucher or Fabry disease were identified, while one patient was diagnosed with glycogen storage disease.

## Discussion

The diagnosis of ASMD type B remains challenging due to its low prevalence, limited recognition, and clinical heterogeneity [2]. To enhance disease identification a consensus guideline has been established by an international panel of experts [2]. Emerging evidence further suggests that lipid abnormalities may serve as potential biomarkers for ASMD, reinforcing the relevance of this criterion in patient identification [2, 7]. Building on this, we first selected patients with a total cholesterol/HDL cholesterol ratio exceeding 4.5, given the high prevalence of dyslipidemia in ASMD type B [4]. Next, we conducted a large-scale analysis of a longitudinal cohort, using an extraction method based on the most frequently reported manifestations in the literature [3]. Our approach successfully identified all pre-existing ASMD type B cases within our cohort. An overrepresentation of individuals of North African origin was observed among both diagnosed ASMD patients (2/3) and those eligible for genetic testing (3/5), in line with previous findings from the French visceral ASMD cohort [8]. The regex-based extraction method proved highly effective in filtering out irrelevant cases and selecting only manifestations consistent with ASMD type B diagnosis. Beyond computational screening, human expertise provided a crucial layer of refinement. A systematic expert review of medical records meeting at least 4 out of 5 diagnostic criteria revealed a wide spectrum of diseases, including undiagnosed cases, highlighting the complementary role of clinical expertise alongside algorithm-based approaches. Our approach differs from previous research, which has mainly used artificial intelligence (AI) methods and data-driven approaches to diagnose ASMD type B within well-defined cohorts or to screen for potential ASMD cases among patients with undiagnosed ILD [9, 10]. Indeed, in a previous study leveraging U.S. healthcare claims data applied a machine learning algorithm to analyze healthcare utilization patterns among patients with ASMD type B [9]. This approach successfully identified potential ASMD type B cases within a cohort of unclassified ASMD patients. Another study utilized machine learning trained on an enriched ASMD cohort, incorporating 199 clinical traits and 11 laboratory measurements [10]. This model achieved ~ 80% sensitivity and > 99% specificity in distinguishing ASMD types A/B or B from matched controls (1:20). When applied to a cohort of 35,930 patients with unexplained interstitial lung disease (ILD), the algorithm flagged 691 potential ASMD patients (< 2%), suggesting that a subset of ILD cases may be linked to undiagnosed ASMD [10]. However, a notable limitation in this study is the lack of detailed clinical characterization of the flagged patients, likely due to the large number of identified cases. This constraint may have limited the ability to assess the algorithm’s specificity and validate its findings in real-world settings.

In our study, the integration of a targeted approach, focusing on key clinical manifestations, with biological data created a strong synergy between data extraction and human expertise. This hybrid methodology effectively addresses some of the inherent challenges of purely algorithmic approaches. Despite certain limitations—such as the retrospective nature of the analysis with missing data, the preselection of patients based on biological criteria, and the underreporting of consanguinity in medical records—the first step, aimed at identifying pre-existing ASMD type B cases, has now been validated. The next step will be to demonstrate the effectiveness of our screening methods by performing genetic screening on identified patients. Notably, five potential candidates for ASMD genetic screening were identified among previously unrecognized cases. Despite these promising findings, an interventional approach with genetic screening remains limited at present due to regulatory constraints related to data protection laws in our study. However, given the significance of our results, efforts are underway to address these challenges. A new ethically approved study is currently in progress to facilitate genetic screening of the identified patients. Then, refining diagnostic criteria may enhance screening sensitivity for distinct subtypes or phenotypically related disorders—for instance, incorporating bone involvement for Gaucher disease or neurological manifestations for ASMD type A. Given the effectiveness of enzyme replacement therapy with olipudase alfa, the recognition and diagnosis of ASMD cases have become particularly critical to ensure timely therapeutic intervention [5, 6].

## Conclusion

Our approach could be expanded to other rare diseases with multi-organ involvement, particularly those with targeted therapies, where detection could significantly improve clinical outcomes for undiagnosed patients. The synergy between data extraction and clinical expertise presents a promising methodology for rare disease diagnosis, enhancing both accuracy and efficiency in patient identification.

## Data Availability

The datasets during and/or analysed during the current study available from the corresponding author on reasonable request.

## Abbreviations

AI: Artificial intelligence
ASMD: Acid Sphingomyelinase Deficiency
CT: Computed Tomography
GGOs: Ground-glass opacities
HDL: High-density lipoprotein
