# Correction: Advanced strategies for detecting acid sphingomyelinase deficiency type B with attenuated phenotypes

**DOI:** 10.1186/s13023-025-03996-7

**Published:** 2025-11-25

**Authors:** Thomas Villeneuve, Thibaut Jamme, Robin Schwob, Thierry Levade, Grégoire Prévot

**Affiliations:** 1 Respiratory Medicine Department, University Hospital, Toulouse, France; 2 Toulouse Institute for Infectious and Inflammatory Diseases (Infinity), INSERM U1291, CNRS U5282, University Toulouse III, Toulouse, France; 3 Clinical Biochemistry Laboratory, Reference Center for Inherited Metabolic Diseases, Federative Institute of Biology, University Hospital, Toulouse, France; 4 Digital and Data Management Department University Hospital, Toulouse, France; 5 Institute of Research in Computer Science of Toulouse - CNRS UMR5505, University Toulouse III, Toulouse, France; 6 Cancer Research Center of Toulouse (CRCT), INSERM UMR1037, University Toulouse III, Toulouse, France

**Correction: Orphanet J Rare Dis 20**,** 252 (2025)**

10.1186/s13023-025-03746-9

Following publication of the original article [1], the authors reported an error in the numerical values of Fig. 1 in the published version.

The incorrect version of Fig. 1 was:

**Fig. 1:**
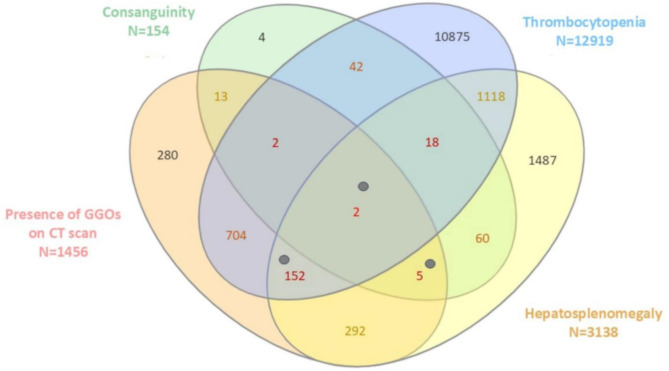
The Venn diagram illustrates the overlap between all types of conditions compatible with ASMD among selected patients with a cholesterol risk ratio > 4.5 n = 63 653). The numbers represent the cohort of patients derived from the total population. Individuals with a confirmed diagnosis of ASMD who met four or five diagnostic criteria are represented by grey points. Abbreviations: CT: Computed Tomography; GGOs: Ground-Glass Opacities

The correct Fig. 1 is:

**Fig. 1:**
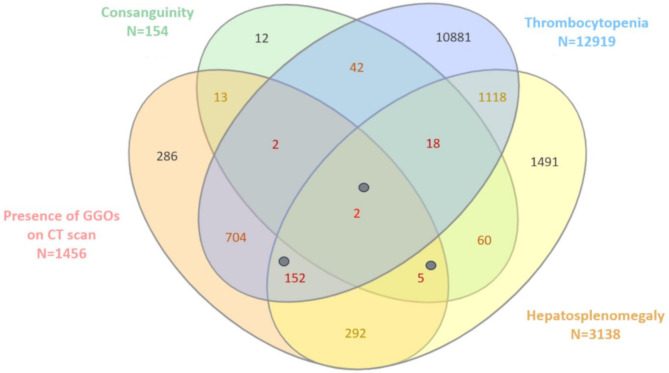
The Venn diagram illustrates the overlap between all types of conditions compatible with ASMD among selected patients with a cholesterol risk ratio > 4.5 n = 63 653). The numbers represent the cohort of patients derived from the total population. Individuals with a confirmed diagnosis of ASMD who met four or five diagnostic criteria are represented by grey points. Abbreviations: CT: Computed Tomography; GGOs: Ground-Glass Opacities

The original article [1] has been updated.
